# Advanced Spectroscopic Characterization of Synthetic Oil from Oil Sands via Pyrolysis: An FTIR, GC–MSD, and NMR Study

**DOI:** 10.3390/molecules30142927

**Published:** 2025-07-10

**Authors:** Ainura Yermekova, Yerbol Tileuberdi, Ainur Seitkan, Anar Gabbassova, Yerlan Zhatkanbayev, Aisha Nurlybayeva, Nurzada Totenova, Stanislav Kotov

**Affiliations:** 1 Department of Chemistry and Chemical Technology, Faculty of Technology, M.Kh. Dulaty Taraz University, Taraz 080000, Kazakhstan; a.yermekova95@gmail.com (A.Y.); rustem_ergali@mail.ru (A.N.); ms.nurzada.93kz@mail.ru (N.T.); 2 Institute of Natural Sciences and Geography, Abai Kazakh National Pedagogical University, Almaty 050010, Kazakhstan; 3 Higher School of Natural Sciences, Astana International University, Astana 010000, Kazakhstan; seitkanainur.77@mail.ru; 4 Department of General and Biological Chemistry, Astana Medical University, Astana 010000, Kazakhstan; 5 Department of Chemistry and Ecology, K. Kulazhanov Kazakh University of Technology and Business, Astana 010000, Kazakhstan; erlan.ntp@mail.ru; 6 Nazarbayev Intellectual Schools, Astana 010000, Kazakhstan; kotov_s@trz.nis.edu.kz

**Keywords:** oil sands, synthetic oil, pyrolysis, FTIR spectroscopy, GC-MSD, NMR spectroscopy

## Abstract

This paper presents a modern spectroscopic characterization of the synthetic oil from oil sands of Beke, Munaily-Mola, and Dongeleksor. The pyrolysis process was carried out at temperatures up to 580 °C with a controlled heating rate, and the products obtained were analyzed using Fourier transform infrared spectroscopy (FTIR), gas chromatography–mass spectrometry (GC–MSD), and nuclear magnetic resonance (NMR) spectroscopy. The FTIR spectra showed a predominance of aliphatic hydrocarbons in the sample from Munaily-Mola synthetic oil, while the content of aromatic compounds was higher in the sample from Beke. GC–MSD analysis revealed significant differences in the distribution of hydrocarbons between the samples, with the Munaily-Mola sample containing a higher proportion of heavy hydrocarbons. NMR spectroscopy provided additional information about the structural composition of the extracted oil. The results indicate the potential of pyrolysis as an effective method for processing oil sands, while the composition of the product varies depending on the geological origin of the raw materials. These findings provide valuable information for optimizing oil sands processing technologies and improving the efficiency of synthetic oil production.

## 1. Introduction

The increasing use of fossil carbon as an energy source is depleting the world’s reserves. As demand for crude oil increases, attention is shifting to alternative energy sources such as oil sands [1,2,3,4]. Oil sands are a valuable resource found in several countries, including Canada, Venezuela, and Kazakhstan, where more than 50 oil sand deposits have been discovered [5,6]. Currently, Kazakhstan is one of the largest oil exporters, and the oil sector plays a significant role in its economy. However, the makeup of natural bitumen (organic part of oil sands or bitumen) allows it to be used as a versatile raw material for making different products, including high-index paraffin oils, high-quality bituminous materials, petroleum sulfides, sulfones, motor fuels, and other products [7,8,9,10,11,12]. The share of conventional oil in the total global reserves [13] is about 30%, while heavy oil accounts for 15%, bitumen from oil sands accounts for about 25%, and extra heavy oil accounts for about 35% [14].

Hot water extraction from oil sands has been a commercial practice for the last several decades [15,16,17]. However, this process creates large volumes of wastewater, which is an environmental problem for the Canadian industry. In this regard, there is a growing interest in more environmentally friendly and cost-effective processing methods, such as pyrolysis, and the thermal decomposition of hydrocarbons at high temperatures.

The pyrolysis of oil sand is a promising method that not only eliminates the use of water and solvents but also improves the quality of bitumen [15]. This method has significant advantages over traditional extraction methods and minimizes environmental risks and costs. Research on the pyrolysis of oil sands fields has demonstrated that heating these materials can be very effective, producing synthetic oil that is similar to regular petroleum products [18,19].

A feature of natural bitumen is its increased density and low oil content with a high resin content [16]. It is present in porous rocks, represented by sands, sandstones, and limestones. The rocks are characterized by weak grain adhesion; they are strongly cemented in sandstones and limestones, which makes it difficult to separate the bitumen. Foreign countries extract natural bitumen using hot water. In our country, this technology is considered energy intensive and inefficient because supplying water to deposits in the West Kazakhstan region poses significant challenges. The extraction of natural bitumen using organic solvents is unprofitable for industry due to the use of costly and hazardous solvents. However, this method can be used in small quantities under laboratory conditions.

In Canada, natural bitumen [20] is processed into “synthetic” oil by slow coking and FlexiCoat processes at Suncor and Syncrude enterprises. However, these processes are energy intensive. They are environmentally unsafe and unprofitable when used at a large scale. The application of the hydrocracking process requires the consumption of hydrogen.

Pyrolysis seems to be a simpler process for industrial production [21]. It does not produce tailings, does not require solvent or water consumption, and can simultaneously improve bitumen during separation. Oil sand can be classified as “oil-wetted” and “water-wetted” [22,23]. Hot water extraction [7] is most effective for oil-soaked sands, such as Buton and Utah oil sands [24]. The phenomenon inspired the search for alternative methods, including pyrolysis, also known as thermal cracking [18,19,25,26,27,28].

Thus, there is sufficient information in the literature on methods for extracting natural bitumen from oil sand, but most are ineffective due to the use of additional expensive and dangerous solvents and reagents. Therefore, this paper proposes pyrolysis as an efficient and economical method for extracting natural bitumen from oil sand. We provide a detailed study using Fourier transform infrared spectroscopy, gas chromatography–mass spectrometry, and nuclear magnetic resonance spectroscopy to analyze synthetic oil (SO).

## 2. Results and Discussion

The compositions of oil sands from the Beke, Munaily-Mola, and Dongeleksor fields during pyrolysis processing were determined. Table 1 presents the experimental results. The sample from the Dongeleksor oil sands had the highest synthetic oil (15.0% by weight) and water content. The largest amount of gas mixture was released from the Munaily-Mola oil sands, which amounted to 1.7. We expected these composition changes to impact the pyrolysis behavior and product distribution.

The composition of the gaseous products includes an organic component derived from the oil sands that remains after pyrolysis (thermal cracking). This organic component enters the liquid products as mechanical impurities. To improve the quality of synthetic oil, it is essential to separate the aqueous part and mechanical impurities. The remaining suspension is then filtered to remove mechanical impurities, resulting in the production of synthetic oil.

Naturally, oil sands are prone to coke formation when exposed to heat. This method of supplying heat with an evaporating agent creates direct contact with oil sands in the reactor, which together slows down the coke formation process and increases the yield of liquid products. As the temperature in the reactor increases, reactions happen that break down large paraffins and naphthenic hydrocarbons into smaller hydrocarbons. Secondary condensation reactions primarily take place in the late stages of pyrolysis processing. As the molecular weight of the reaction product molecules increases, the gaseous volume of the reaction mass decreases. The creation of aromatic and polynuclear aromatic hydrocarbons, like naphthalene, anthracene, resins, and asphaltenes, from the condensation reaction results in the production of heat-resistant aromatic hydrocarbons, such as coke and petroleum pitch. To slow down the secondary thermolysis reactions, oil sands from pyrolysis processing are mixed with an evaporative agent at temperatures of 400–450 °C. As a result, the partial pressure of hydrocarbons decreases; according to the Le Chatelier principle, a decrease in pressure in the reaction zone will promote reactions that favor primary reactions. It is proposed to increase the selectivity of the process and enhance product yield during pyrolysis processing by reducing the residence time of the evaporating agent’s gas in the reaction zone and raising the reactor’s temperature.

### 2.1. Infrared Spectroscopic Study of the Synthetic Oil

**Figure 1:**
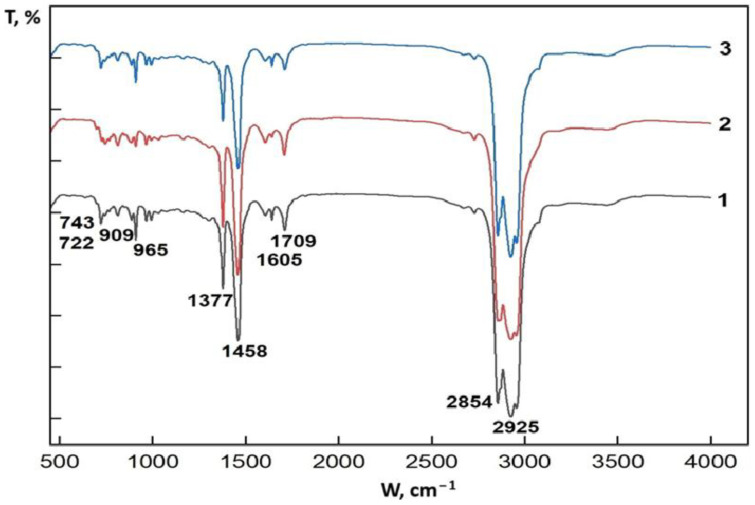
FTIR spectra of synthetic oil produced from oil sands of the Beke (1), Munaily-Mola (2), and Dongeleksor (3) deposits.

Infrared absorption spectra of the synthetic oil from oil sand samples were studied using a Fourier transform infrared spectrometer in the range of 400–4000 cm^−1^. FTIR spectroscopy was employed to study the composition of substances by detecting functional groups or bonds in molecules along the corresponding absorption bands. Figure 1 shows the FTIR spectra of the liquid fractions obtained from each sample.

From the spectra of three samples, we found specific absorption bands that match aliphatic, aromatic, and oxygen-containing compounds. A comparative analysis of the purification degree and composition of the oil samples was carried out. Oil sand synthetic oil is a complicated mix of hydrocarbons that have different weights and structures, including types like aliphatic, aromatic, and naphthenic compounds, along with other elements that contain oxygen, nitrogen, and sulfur. One of the best ways to study what petroleum is made of is using FTIR (Fourier Transform Infrared) spectroscopy, which helps identify different functional groups by looking at their unique absorption patterns.

The strong absorption bands at 2925–2854 cm^−1^ correspond to aliphatic C-H stretching fluctuations, which were most intense in the Munaily-Mola sample. The 1605 cm^−1^ band associated with the stretching of aromatic C=C bonds was noticeable in the Beke sample, indicating a higher concentration of polycyclic aromatic hydrocarbons. The presence of a 1709 cm^−1^ band (elongation C=O) in all samples indicates partial oxidation of hydrocarbons during pyrolysis [18,29].

These results show that synthetic oil produced from the oil sand fields of Munaily-Mola contains a higher proportion of saturated hydrocarbons, while Beke produces more aromatic compounds that can affect the viscosity and stability of the final product. The same absorption bands are observed in the spectra of synthetic oil from the oil sands of all three deposits, which were analyzed in the range of 4000 to 500 cm^−1^ to compare their intensities. The functional groups identified by absorption bands and their intensity values are presented in Table 2.

Sample 1 Beke synthetic oil: It has the most functional groups, including carbonyl compounds (1709 cm^−1^), aromatic rings, and long aliphatic chains. It probably represents an untreated or heavy fraction of oil with a high content of resins and asphaltenes.

Sample 2 Munaily-Mola synthetic oil: It contains the main characteristics of oil, but with a lower intensity of carbonyl bands, which indicates partial purification.

Sample 3 Dongeleksor synthetic oil: the most “pure” spectrum mainly shows aliphatic hydrocarbons, with very few oxygen-containing compounds. It can correspond to light fractions, for example, gasoline or kerosene.

The results of the FTIR spectroscopic analysis show a relatively high content of aliphatic compounds in the synthetic oil from the Munaily-Mola deposit, while the content of aromatic and oxygen-containing compounds in the other synthetic oil was found to be the same.

### 2.2. GC–MSD Analysis of the Composition of Synthetic Oil

Gas chromatography–mass spectrometry (GC–MSD) provided detailed information on the molecular composition of pyrolysis products. The synthetic oil from oil sands of Beke, Munaily-Mola, and Dongeleksor is made up of a variety of natural substances that include different types of hydrocarbons and polar compounds, such as asphaltenes, resins, and both aliphatic and aromatic hydrocarbons. To thoroughly analyze the chemicals and separate the different parts, we used adsorption chromatography methods, especially with columns filled with alumina and silica gel.

The role of alumina and silica gel in adsorptive separation is significant. Alumina is a polar adsorbent with a high sorption capacity for polar and high-molecular-weight compounds such as resins and asphaltenes. During the passage of synthetic oil from oil sand samples through a column packed with alumina, the heavy polar components are adsorbed and retained on the surface of the adsorbent, allowing for effective separation from less polar and non-polar hydrocarbons.

Prior to GC–MSD analysis, the synthetic oil underwent preparative purification and fractionation. The samples were passed through sequential adsorption columns filled with alumina and silica gel, respectively. Using adsorbents that attract different types of molecules helped to keep back certain polar, heavy components (like asphaltenes and resins), which made the chromatographic data more accurate and consistent. This pre-treatment step efficiently fractionated the hydrocarbon classes—aliphatic, cyclic, olefinic, and aromatic compounds—while also reducing the matrix interferences in GC–MSD analysis. Before analyzing the synthetic oil, samples from Beke, Munaily-Mola, and Dongeleksor were first prepared by passing them through a series of adsorption columns filled with alumina and silica gel. The process of passing synthetic oil from oil sand samples through adsorption columns involves several steps: Passing the sample through the alumina column primarily removes and retains heavy polar compounds—such as asphaltenes and resins—that can significantly affect the accuracy and consistency of the analytical data; afterward, the sample passes through the silica gel column, which facilitates the separation of aliphatic hydrocarbons, alkenes, aromatic compounds, and other classes of substances.

As a result of sequential passage through the two adsorption columns, the synthetic oil from the oil sand material sample was purified and fractionated into distinct components, which were then subjected to further chromatographic analysis—such as gas chromatography with mass spectrometric detection.

Using adsorption columns filled with alumina and silica gel to analyze synthetic oil from oil sand samples from Beke, Munaily-Mola, and Dongelek Sor is an important step in preparing the samples, which ensures

- Effective removal of heavy and polar components (such as asphaltenes and resins) that may interfere with accurate analysis;
- Clear separation of the petroleum mixture into classes of hydrocarbons and other organic compounds;
- Enhanced sensitivity and reproducibility of chromatographic analysis;
- More comprehensive and reliable information about the chemical composition of synthetic oil from oil sand.

**Figure 2:**
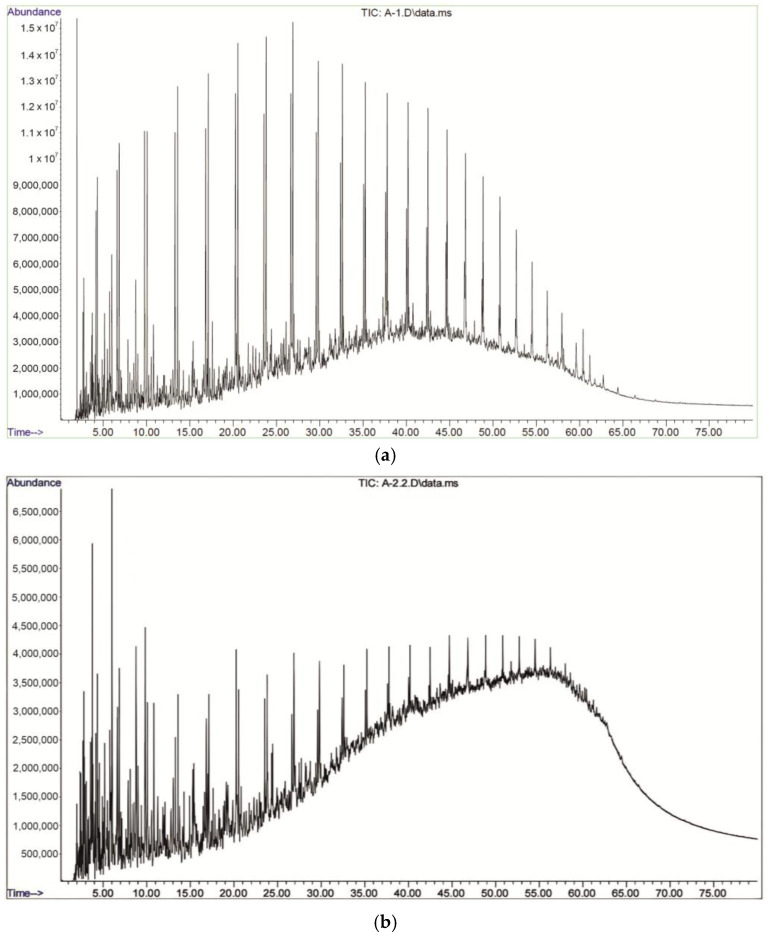

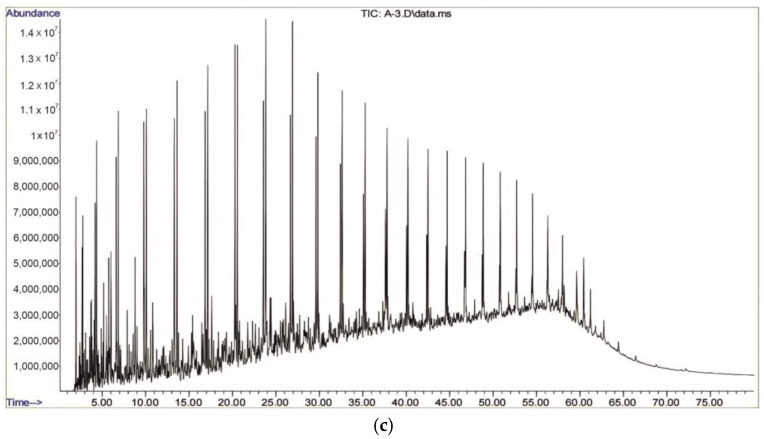
Chromatograms of synthetic oil from the Beke (**a**), Munaily-Mola (**b**), and Dongeleksor (**c**) oil sand deposits.

**Figure 3:**
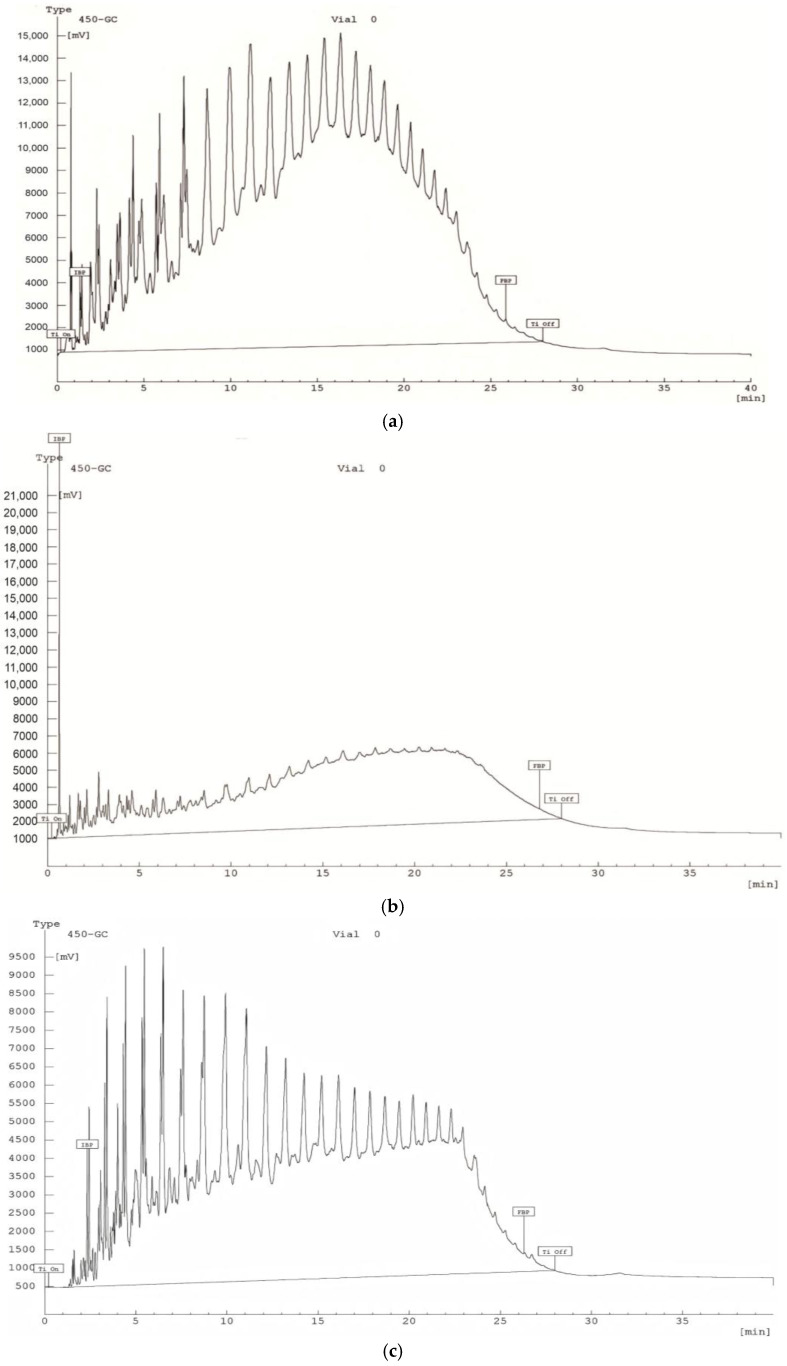
Gas chromatography–mass spectrometry of SO from the Beke (**a**), Munaily-Mola (**b**), and Dongeleksor (**c**) oil sand deposits.

Table 3 presents only the major peaks corresponding to compounds with the highest intensities, selected to improve readability and focus on key components. The full chromatographic profiles, including minor mass fractions and the groups (up to 35 min), are shown in Figure 2 and Figure 3. The chromatograms show significant differences in the distribution of hydrocarbons between the samples.

A higher concentration of low-molecular-weight hydrocarbons (C_6_–C_12_) with dominant peaks in the mass fraction of 5–20 min was found in the Beke (a) sample. The Munaily-Mola (b) sample contained hydrocarbons with a higher molecular weight (C_15_-C_30_), which indicates a heavier fractional composition. The Dongeleksor (c) sample showed a balanced distribution of hydrocarbons, which indicates the presence of a mixture of aliphatic and aromatic components.

The presented data show the mass fractions of various hydrocarbons and other organic compounds in the synthetic oil from the oil sand sample. The sample mainly has straight-chain alkanes, ring-shaped alkanes, alkenes, and aromatic compounds, along with some specific organic materials like ketones, alcohols, and lactones.

These results not only describe the crude oil’s overall composition but also suggest that an adsorption column purified the sample. Adsorption columns, typically based on alumina and silica gel, are designed to remove heavy polar components—such as asphaltenes—as is evident from the results:

- Asphaltenes and resins are absent or present in extremely low concentrations, as these heavy, high-molecular-weight compounds are retained in the adsorption column. This method enables the separation of crude oil into lighter and medium-molecular-weight fractions.
- The composition is predominantly characterized by aliphatic alkanes (e.g., *n*-hexane, heptane, octane, nonane), aliphatic cycloalkanes (e.g., derivatives of cyclopentane and cyclohexane), alkenes (e.g., 2,4,4-trimethyl-1-hexene, 1-heptene, 2-octene), and aromatic compounds (e.g., toluene, *p*-xylene).

In addition, small amounts of alcohols, ketones, lactones, azetidine, and other organic compounds were detected in the synthetic oil composition.

These results confirm that the geological origin of oil sands significantly affects the composition of the produced synthetic oil. The Munaily-Mola fraction is more suitable for the production of diesel fuel, while the Beke fraction with its higher content of aromatic substances may be better suited for the production of bitumen and heavy fuels.

Gas chromatographic analysis with mass spectrometric detection allowed us to determine the hydrocarbon composition of the samples studied [30]. The chromatograms of the Beke sample show peaks in the range of 5–20 min, which indicates a high content of light and medium hydrocarbons (C_6_–C_12_). In the Munaily-Mola sample, the main elution occurs in the 20–30 min interval, which is typical for heavier fractions (C_15_–C_30_) rich in asphaltenes and resins. A uniform distribution of fractions in the range of 10–35 min is observed in the Dongeleksor sample, which indicates a balanced composition.

### 2.3. Analysis of the Structural Composition of the Synthetic Oil by NMR Spectroscopy

**Figure 4:**
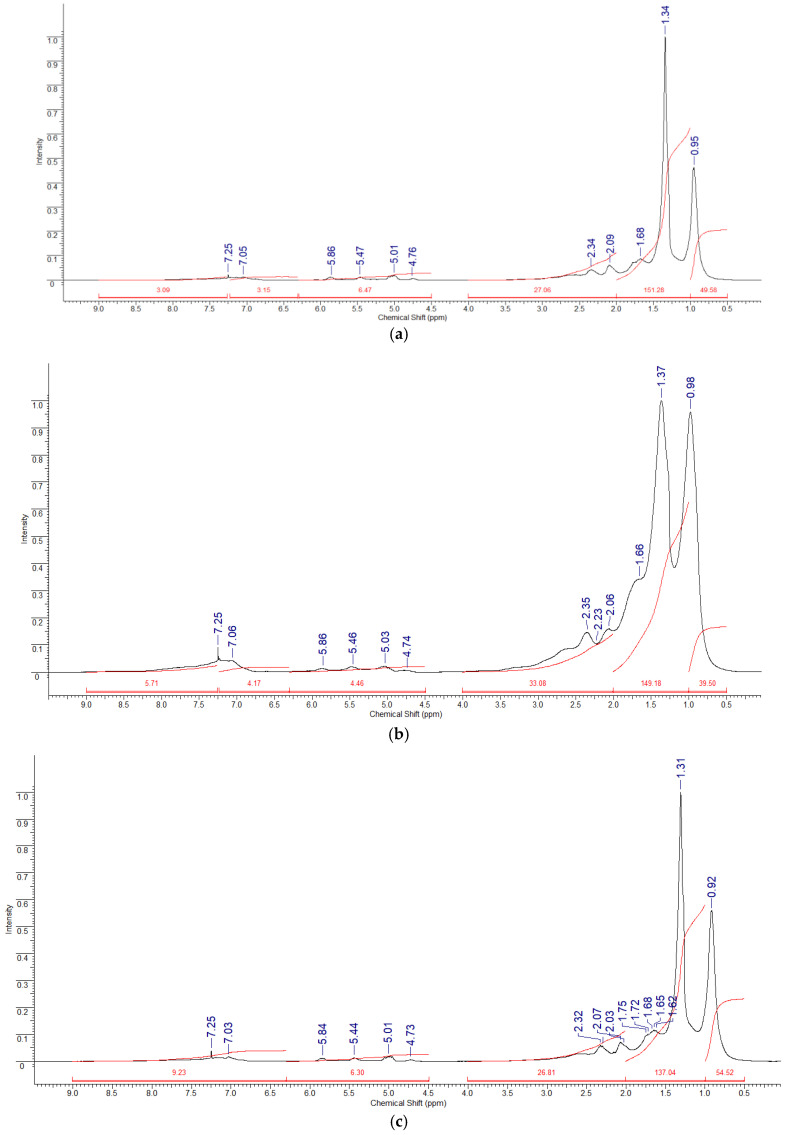
^1^H NMR spectra of samples dissolved in SDKl_3_: Beke (**a**), Munaily-Mola (**b**), and Dongeleksor (**c**).

NMR spectroscopy was used to determine the molecular structure of the pyrolysis products of the Beke, Munaily-Mola, and Dongeleksor samples. The ^1^H NMR spectra (Figure 4) allowed us to estimate the distribution of hydrogen atoms in various chemical media. Munaily-Mola synthetic oil showed strong signals in the range of 0.5–2.0 ppm, corresponding to aliphatic protons (–CH_3_, –CH_2_–), indicating a high content of saturated hydrocarbons. The oil obtained from Beke showed intense peaks in the range of 6.3–9.0 ppm associated with aromatic protons, which confirms the presence of polycyclic aromatic hydrocarbons. Dongeleksor oil demonstrated a balanced distribution of aliphatic and aromatic protons with signals in the ranges of 0.5–2.5 ppm (alkyl groups) and 6.5–8.0 ppm (aromatic protons), indicating a mixed composition of saturated and unsaturated hydrocarbons.

**Figure 5:**
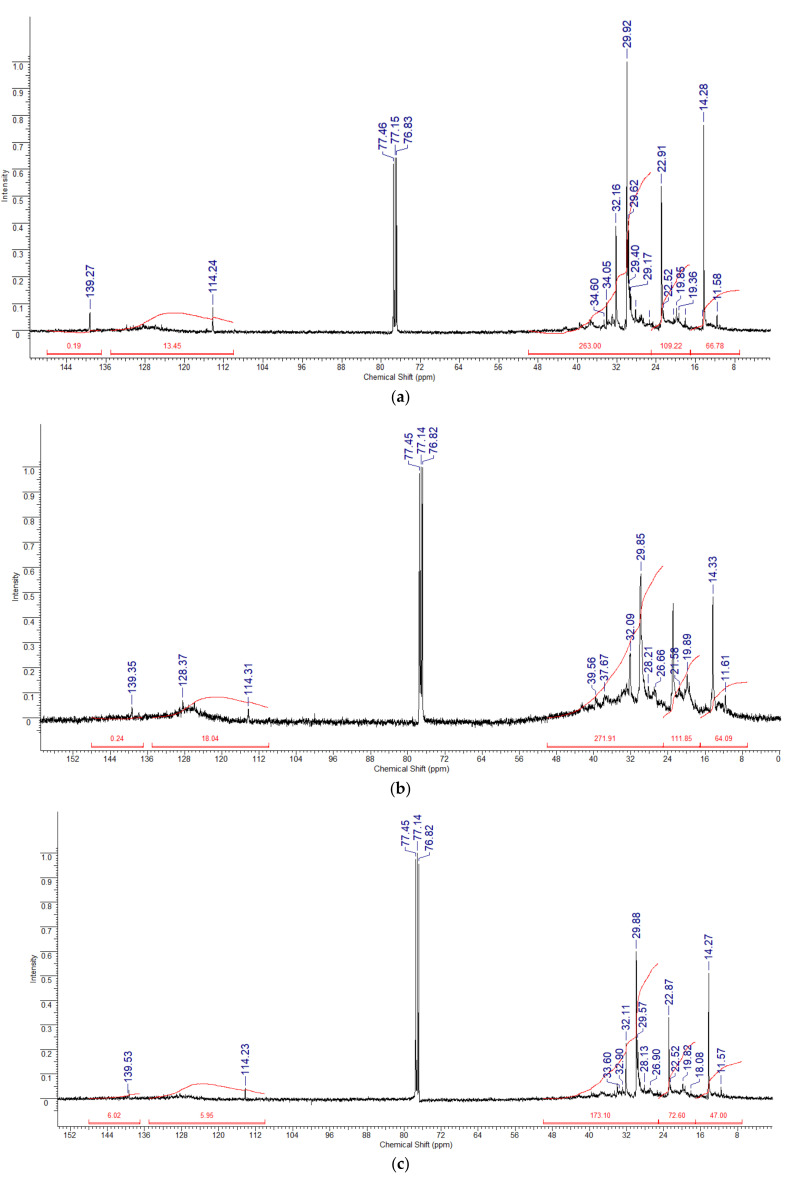
^13^C NMR spectra of samples dissolved in CDCl_3_: Beke (**a**), Munaily-Mola (**b**), and Dongelekor (**c**).

The ^13^C NMR spectra (Figure 5) further confirmed these results: The Munaily-Mola sample contained a higher proportion of aliphatic carbon signals (10–50 ppm), which confirms the predominance of linear and branched paraffins. A high content of aromatic hydrocarbons (110–160 ppm) was found in the Beke sample, which corresponds to a high content of aromatic substances. The Dongeleksor sample showed a combination of aliphatic and aromatic carbon signals, indicating a mixture of paraffin and aromatic hydrocarbons. The presence of signals in the range of 50–80 ppm indicates the presence of naphthenic structures that may affect the intermediate properties of this sample.

^1^H and ^13^C NMR spectroscopy enabled us to characterize the hydrocarbon nature of the studied samples. The ^1^H NMR spectra for Munaily-Mola synthetic oil are dominated by signals in the range of 0.5–2.0 ppm, corresponding to saturated aliphatic compounds, confirming its paraffin nature. In the Beke samples, the most intense peaks were recorded in the range of 6.3–9.0 ppm, indicating a high content of aromatic compounds. The ^13^C NMR spectra showed the presence of saturated aliphatic hydrocarbons (10–50 ppm) and aromatic hydrocarbons (110–160 ppm), with the latter being more pronounced in the Beke samples. Thus, the Munaily-Mola sample is characterized by a high content of aliphatic hydrocarbons, making it suitable to produce diesel fuel and oils, while the Beke sample contains more aromatic components, positioning it as a promising raw material for bitumen production. Integration of ^1^H and ^13^C spectra allowed calculation of the proportion of saturated, aromatic, and olefinic structures. The presence of branched hydrocarbons suggests high octane potential.

According to the methods recommended in [31], the limits of chemical shifts of the proton and carbon spectra with their corresponding interpretations are given in Table 4 and Table 5. In this study, the NMR spectra of hydrogen ^1^H and carbon ^13^C nuclei dissolved in deuterated chloroform were taken on a JNM-ECA Jeol 400 spectrometer at a temperature of 25 °C. The operating frequencies for cores ^1^H and ^13^C were assumed to be 399.78 MHz and 100.53 MHz, respectively. Chemical shifts of δ were measured relative to the signals of residual protons or carbon atoms of deuterated chloroform.

Even though proton-decoupled ^13^C NMR spectra are not completely accurate because of differences in relaxation times (T_1_) and the nuclear Overhauser effect (NOE), many recent studies have shown that this method works well for estimating the structure of complicated hydrocarbon systems, like pyrolysis oils and petroleum products [31,32].

The total integral intensity of the ^1^H signals is calculated using the following formula:

(1) $${\bar{H}}_{sum}=H_{ar}\;+\;H_{\alpha }\;+\;H_{\beta }\;+\;H\gamma ,$$

There are $H_{ar}$, $H_{\alpha }$, $H_{\beta }$, $H_{\gamma }$—integral intensities of aromatic protons and protons at the *α*, *β*, and *γ* positions in the aromatic ring, respectively.

The percentages of aromatic and aliphatic protons are respectively determined by the following formulas:

(2) $${\bar{H}}_{ar}=\frac{H_{ar}}{{\bar{H}}_{sum}}\;\cdot 100\;\%,$$

(3) $${\bar{H}}_{al}=\;\frac{H_{\alpha }\;+\;H_{\beta }\;+\;H_{\gamma }}{{\bar{H}}_{sum}}\cdot 100\;\%.$$

The percentages of protons in the *α*, *β*, and *γ* positions of the aromatic ring are found, respectively, by the following formulas:

(4) $${\bar{H}}_{\alpha }=\frac{H_{\alpha }}{{\bar{H}}_{sum}}\cdot 100\%,\;\;{\bar{H}}_{\beta }=\frac{H_{\beta }}{{\bar{H}}_{sum}}\cdot 100\%,\;\;{\bar{H}}_{\gamma }=\frac{H_{\gamma }}{{\bar{H}}_{sum}}\cdot 100\%.$$

In this study, the amounts of important carbon types—aliphatic, aromatic, and olefinic—were estimated by looking at the signal strengths in specific parts of the ^13^C spectrum (for example, 10–50 ppm for saturated aliphatic carbons and 110–160 ppm for aromatic carbons). To make sure the data could be compared and repeated, all the spectra were collected using the same experimental setup: 1024 scans, the same pulse sequences, and set relaxation delay times. Samples were dissolved in CDCl_3_ at a controlled concentration (~30 mg/0.6 mL) and analyzed at 25 °C.

This semi-quantitative approach provides a rough estimate that follows standard procedures and matches newer developments in analyzing oils made from heat treatment using NMR [31,32]. While absolute quantification remains limited, relative comparisons between samples are valid when acquisition parameters are controlled. The results provide useful information about the distribution of carbon types in synthetic oils derived from different oil sands, supporting their structural characterization and potential for downstream processing (Table 6).

There are no water protons in this sample. The ^13^C NMR spectra of the oil sample show that the proton spectra correlate with the carbon spectra. The NMR carbon spectra are located in the range of 11.58–139.27 ppm. The ratios of the integral intensities of carbon atoms Sp, H (primary carbon atoms with a methylene group), Sp (primary carbon atoms associated with the CH group or aromatic core), and Cb+h (secondary and quaternary atoms with saturated compounds) are presented in the table. It should be noted that the greatest integral intensity of carbon atoms is observed in the region of 25–50 ppm, corresponding to the region of quaternary carbon atoms of saturated compounds. This indicates a high degree of branching hydrocarbon chains and a possible high degree of anti-knock properties of hydrocarbon fuels based on it.

## 3. Materials and Methods

### 3.1. Raw Materials and Supplies

Oil sand samples were collected from three fields in Kazakhstan: Beke oil sands from Mangystau region, Munaily-Mola oil sands from Atyrau region, and Dongeleksor oil sands from Aqtobe region. The samples were dried to remove residual moisture and then ground to a uniform particle size of <2 mm for experimental analysis. Prior to pyrolysis, we determined the moisture content using a muffle oven at 105 °C.

### 3.2. Pyrolysis Procedure

The processing of oil sand was conducted under laboratory conditions with a consistent heating rate from room temperature to 580 °C. The heating rate used was 20 °C per minute. Each experiment involved placing 300 g of oil sand in a vertical reactor and heating it without the use of catalyst and carrier gas. The pyrolysis process continued until no more oil drops came out. The average duration of the process was 50 min. It measured the solid residue after the pyrolysis procedure to determine the amount of conversion.

### 3.3. Fourier Transform Infrared Spectroscopy (FTIR)

Functional groups in the liquid pyrolysis products were identified using a Fourier transform infrared spectrometer (FTIR) (Nicolet iS50, Thermo Fisher Scientific, Waltham, MA, USA). The spectra were recorded in the 4000–400 cm^−1^ range with a resolution of 4 cm^−1^ using the attenuated total reflection (ATR) method.

### 3.4. Gas Chromatography–Mass Spectrometry (GC–MSD)

The composition of pyrolysis oil hydrocarbons was analyzed using a GC–MSD system (Agilent 7890A GC in combination with the Triple-Axis MSD 5975C detector, Agilent Technologies, Santa Clara, CA, USA). Separation was performed on an HP-5MSD capillary column (30 m × 0.25 mm × 0.25 µm, Agilent Technologies, Santa Clara, CA, USA) with helium as the carrier gas (flow rate: 1.0 mL/min). The injection temperature was set to 280 °C, and the column temperature was programmed as follows: an initial temperature of 40 °C for 2 min, an increase of 4 °C/min to 290 °C, followed by a hold for 30 min. The MSD detector operated in scanning mode (*m*/*z* 50–600).

### 3.5. Nuclear Magnetic Resonance (NMR) Spectroscopy

The structural characteristics of pyrolysis oil were obtained using ^1^H and ^13^C NMR spectroscopy on a JNM-ECA Jeol 400 MHz spectrometer (JEOL Ltd., Tokyo, Japan). The samples were dissolved in deuterated chloroform (CDCl_3_; Sigma-Aldrich, St. Louis, MO, USA), and chemical shifts (δ) were calculated relative to the peak of the residual solvent (7.26 ppm for ^1^H, 77.16 ppm for ^13^C). The spectra were recorded at 25 °C with 64 scans for ^1^H NMR and 1024 scans for ^13^C NMR. Water was not observed in the organic phase in the ^1^HMR spectra, since the aqueous phase had been separated prior to analysis.

### 3.6. Statistical Analysis

All quantitative results were subjected to statistical analysis using GraphPad Prism software (version 5.0; GraphPad Software Inc., La Jolla, CA, USA). To compare the average values of the groups, we used one-way analysis of variance (ANOVA) and then Duncan’s multiple range test to find any significant differences, with a significance level set at *p* < 0.05. The data are presented as the means ± standard deviations (SDs) calculated from three separate experimental replicates.

## 4. Conclusions

The analysis of the obtained products shows satisfactory parameters according to the standards for synthetic crude oil. The experiments began with the release of hydrocarbon gases and water from the oil sand samples. However, the presence of water indicated a potential reduction in processing complexity. During the processing of oil sand, at a certain temperature, product formation reached a peak and then leveled off. The required temperature was not very energy efficient (about 580 °C).

Studies using complex methods like IR spectroscopy, GC–MS, and NMR have revealed that oils from different sources have clear differences in their composition and physical–chemical properties. Compounds found in the structure of oil sand can expedite the process without the need for expensive catalysts. The synthetic oil produced during this process can be utilized in various fields, such as gasoline production or chemical synthesis.

IR-Fourier spectroscopy has demonstrated its effectiveness in analyzing the composition of oil. The revealed absorption bands allowed us to judge the presence of aliphatic, aromatic, and oxygen-containing compounds. Comparing the spectra of different samples provided us an idea of their degree of purification and chemical composition, which is important for further refining and use of oil in various industries.

Using columns made of alumina and silica gel helped us effectively study the complicated makeup of synthetic oil from oil sand materials. This capacity is essential both for fundamental scientific research and for practical tasks related to the processing and utilization of hydrocarbon resources.

Munaily-Mola synthetic oil is rich in aliphatic hydrocarbons and is characterized by high thermal stability, making it suitable to produce lubricants and heavy fuels. Beke oil contains a significant amount of aromatic compounds and light fractions, making it promising to produce gasoline and bitumen. Dongeleksor occupies an intermediate position, combining characteristics from both groups, which makes it a versatile candidate for processing. These results can be used to optimize oil refining processes and select the most effective methods for their industrial use.

Thus, this study represents a significant contribution to the field of pyrolysis of oil sands and can serve as a foundation for further research aimed at optimizing pyrolysis processes and enhancing product quality. A comparison of different types of oil sands, such as Munaily-Mola, Beke, and Dongeleksor, offers a more comprehensive understanding of the possibilities and limitations of pyrolysis for various types of hydrocarbons.

## Figures and Tables

**Table 1:** Oil sand compositions after pyrolysis.

№	Oil Sand Samples	Synthetic Oil, wt.%	Mineral Part, wt.%	Separated Gases, wt.%	Water Content, wt.%
1	Beke	10.2 ± 0.3	88.0 ± 0.3	1.5 ± 0.1	0.3 ± 0.01
2	Munaily-Mola	13.9 ± 0.3	84.0 ± 0.3	1.7 ± 0.1	0.4 ± 0.01
3	Dongeleksor	15.0 ± 0.4	83.0 ± 0.4	1.6 ± 0.1	0.6 ± 0.02

**Table 2:** FTIR spectra of synthetic oil obtained by pyrolysis of oil sands.

Wavenumber (cm^−1^)	Band Assignment	Corresponding Functional Group	Interpretation
2925, 2854	Valence vibrations of C–H	–CH_2_, –CH_3_	Aliphatic hydrocarbons (paraffins)
1709	Valence vibrations of C=O	Carbonyl group	Oxygen-containing compounds: ketones, acids, resinous components
1605	Fluctuations of C=C in the aromatic ring	Aromatic hydrocarbons	Benzene and naphthalene derivatives
1458, 1377	Deformation vibrations –CH_3_ and –CH_2_	Methyl and methylene groups	Aliphatic chains
965, 909, 743, 722	Deformation vibrations C–H	Aromatic and cycloalkanes	Indicate the presence of rings, naphthenes, aromatic structures

**Table 3:** Chromatograms of synthetic oil from oil sands.

№	Mass Fraction, %	Compound Name	Groups
1	2	3	4
1	0.04	2,4,4-Trimethyl-1-hexene	Alkenes
2	0.72	n-Hexane	Aliphatic alkanes
3	0.07	Cyclopentane, methyl	Aliphatic cycloalkanes
4	0.19	Cyclopentene, methyl	Alkenes
5	0.04	2(3H)-Furanone, dihydro-5-methyl	Others (furanone, lactone)
6	0.10	Hexane, 3-methyl	Aliphatic alkanes
7	0.29	1-Heptene	Alkenes
8	0.32	Heptane	Aliphatic alkanes
9	0.53	Cyclopentene, 4,4-dimethyl	Alkenes
10	0.30	Cyclohexane, methyl	Aliphatic cycloalkanes
11	0.07	Cyclopentane, ethyl	Aliphatic cycloalkanes
12	0.46	Cyclohexene, 4-methyl	Alkenes
13	0.02	Cyclobutanone, 2,3,3-trimethyl	Others (ketones)
14	0.02	1,3,5-Hexatriene, 3-methyl	Alkenes
15	0.12	2,4-Heptadiene, (E,E)	Alkenes
16	0.42	Heptane, 2-methyl	Aliphatic alkanes
17	0.24	Toluene	Aromatic compounds
18	0.08	Cyclohexane, 1,2-dimethyl	Aliphatic cycloalkanes
19	0.17	1-Heptene, 2-methyl	Alkenes
20	0.59	2-Octene	Alkenes
21	0.69	Octane	Aliphatic alkanes
22	0.24	3-Octene	Alkenes
23	0.32	Cyclopentene, 1,2,3-trimethyl	Alkenes
24	0.22	Cyclohexane, ethenyl	Aliphatic cycloalkanes
25	0.10	Bicyclo[3.1.0]hexane, 1,5-dimethyl	Aliphatic cycloalkanes
26	0.04	Cyclohexanol, 2,6-dimethyl	Others (alcohols)
27	0.63	Cyclohexane, 1,1,3-trimethyl	Aliphatic cycloalkanes
28	0.12	Cyclohexene, 1,6-dimethyl	Alkenes
29	0.05	1-Octene, 3-methyl	Alkenes
30	0.26	1-Heptene, 2,6-dimethyl	Alkenes
31	0.08	Nonane, 4-ethyl-5-methyl	Aliphatic alkanes
32	0.23	Cyclopentane, 2-ethylidene-1,1-dimethyl	Aliphatic cycloalkanes
33	0.20	Cyclohexane, 1,2,3-trimethyl	Aliphatic cycloalkanes
34	0.33	*p*-xylene	Aromatic compounds
35	0.05	Ethylidenecycloheptane	Aliphatic cycloalkanes
36	0.15	2,4-Dimethyl-1-heptene	Alkenes
37	0.13	Azetidine-2-one, 3-hexyl-3-methyl	Others (azetidine)
38	0.04	Cyclohexane, 1-ethyl-2-methyl	Aliphatic cycloalkanes
39	1.07	1-Nonene	Alkenes
40	0.04	Cyclohexene, 3,3,5-trimethyl	Alkenes
41	1.20	Nonane	Aliphatic alkanes
42	0.10	2-Nonene	Alkenes
43	0.09	1-Octene, 3,7-dimethyl	Alkenes
44	0.03	cis-2-Nonene	Alkenes
45	0.07	1,3-Hexadiene, 3-ethyl-2-methyl	Alkenes
46	0.03	Cyclohexane, 2-propenyl	Aliphatic cycloalkanes
47	0.07	4-Nonyne	Alkynes
48	0.08	Cyclohexane, propyl	Aliphatic cycloalkanes
49	0.14	Octane, 2,6-dimethyl	Aliphatic alkanes
50	0.05	3,6-Nonadien-1-ol	Others (alcohols)

**Table 4:** Limits of change and interpretation of chemical shifts of ^1^H nuclei.

δ(^1^H) ppm	Atom Designation	Functional Group
0.5–1.0	H_γ_	CH_3_ groups of saturated compounds. CH_3_ groups in *γ*- and further positions to the aromatic ring
1.0–2.0	H_β_	CH_2_ and CH groups of saturated compounds. Hydrogen atoms of *β*-methyl, *β*- and further methylene and methine groups at the aromatic ring
2.0–4.0	H_α_	Hydrogen atoms in the α-position to aromatic and carbonyl carbons, heteroatoms
4.5–6.3	H_olef_	Hydrogen atoms of olefin groups
6.3–9.0	H_ar+ph_	Hydrogen atoms of aromatic rings, phenolic hydroxyls

**Table 5:** Change limits and interpretation of chemical shifts of ^13^C nuclei.

δ(^13^C) ppm	Atom Designation	Functional Group
7–17	C_p,m_	Primary carbon atoms at the methylene group
17–25	C_p,ar_	Primary carbon atoms bonded to the CH group or aromatic ring
17–50	C_(sec+quart)_	Secondary and quaternary C atoms of saturated compounds
25–65	C_alip_	Aliphatic CH groups
25–50	C_quar_	Quaternary carbon atoms of saturated compounds
108–118	C_olef_	Olefin fragments
110–135	C_ar_	Tertiary C atoms of aromatic systems
130–137	C_m, ar_	Methyl-substituted aromatic carbon atoms
137–148	C_(ar+alkyl)_	Alkyl- and naphthyl-substituted carbon atoms of aromatic rings
148–170	C_ar_	Aromatic carbon atoms substituted by a phenol or ether group
170–200	C_carb_	Carbonyl carbon atoms

**Table 6:** Fragment composition according to ^1^H NMR spectra (% mass) and ^13^C NMR spectra (% mass).

Type of Atoms	Synthetic Oil Beke	Synthetic Oil Munaily-Mola	Synthetic Oil Dongeleksor
H_ar_	2.6	4.2	3.9
H_alip_	97.4	95.8	96.1
H_α_	11.2	14.0	11.5
H_β_	62.9	63.2	58.6
H_γ_	20.6	16.7	23.3
H_olef_	2.7	1.9	2.7
C_p,m_	14.8	13.7	12.8
C_ar_	3.7	3.9	1.6
C_(sec+quart)_	82.2	82.3	47.7
C_(ar+alkyl)_	0	0.1	37.9

Values are mean ± SD (*n* = 3). The *p*-value results are as follows: (*p* < 0.05).

## Data Availability

The original results presented in this study are included in the article. Further inquiries can be directed to the corresponding author.
